# Postpartum during a pandemic: Challenges of low-income individuals with healthcare interactions during COVID-19

**DOI:** 10.1371/journal.pone.0268698

**Published:** 2022-05-24

**Authors:** Maria V. Gomez-Roas, Ka’Derricka M. Davis, Karolina Leziak, Jenise Jackson, Brittney R. Williams, Joe M. Feinglass, William A. Grobman, Lynn M. Yee

**Affiliations:** 1 Division of Maternal-Fetal Medicine, Department of Obstetrics and Gynecology, Northwestern University Feinberg School of Medicine, Chicago, Illinois, United States of America; 2 University of Michigan Medical School, Ann Arbor, Michigan, United States of America; 3 Division of General Internal Medicine and Geriatrics, Department of Medicine, Department of Preventive Medicine, Northwestern University Feinberg School of Medicine, Chicago, Illinois, United Satates of America; Kyushu University, JAPAN

## Abstract

**Background:**

Changes to the healthcare system due to COVID-19 have altered care delivery during birth and the postpartum period, a transitional time that requires intensive healthcare support and that is complicated by well-established health disparities. Our objective was to identify additional challenges to healthcare interactions that emerged for low-income postpartum individuals during the pandemic.

**Methods:**

This is a qualitative investigation of low-income postpartum individuals enrolled in a trial of postpartum care, who gave birth in the United States in the first three months of the COVID-19 pandemic. Participants completed in-depth semi-structured interviews that addressed healthcare experiences during and after birth, both for in-person and telemedicine encounters. Transcripts were analyzed using the constant comparative method.

**Results:**

Of 46 eligible individuals, 87% (N = 40) completed an interview, with 50% identifying as non-Hispanic Black and 38% as Hispanic. Challenges were organized into three domains: unanticipated changes in the birth experience, delayed care, and perceived disadvantages of telemedicine. Changes in the birth experience addressed uncertainty about COVID-19 status, COVID-19 testing, separation from newborn, and visitor restrictions. Delayed care themes addressed logistical challenges, postpartum care, health maintenance, and pediatric care. Participants reported multiple telemedicine-related challenges, including difficulty establishing rapport with providers.

**Conclusions:**

Understanding the challenges experienced by low-income peripartum individuals as the COVID-19 pandemic evolves is critical to informing guidelines and diminishing inequities in healthcare delivery. Potential solutions that may mitigate limitations to care in the pandemic include emphasizing shared decision-making in care processes and developing communication strategies to improve telemedicine rapport.

## Introduction

The postpartum period is a challenging phase during which individuals are recommended to receive intensive psychosocial and health care support as they transition from pregnancy to parenthood, while recovering from giving birth and caring for themselves and their newborns. Healthcare engagement during this period is an essential component of promoting short- and long-term health outcomes. Yet, postpartum care in the United States (US) remains highly fragmented, with as many as 40% of individuals not attending a postpartum care visit, and with well-established disparities that undermine the delivery of optimal care during this period [1]. As a result, the US Department of Health and Human Services’ (DHHS) Healthy People 2020 goals included increasing postpartum visit attendance as one of its priorities, a goal echoed by many professional organizations [2].

As the COVID-19 pandemic reshapes the delivery of healthcare, existing barriers have been exacerbated, further restricting how pregnant and postpartum individuals access care and interact with the healthcare system. Moreover, guidelines aiming to reduce the spread of COVID-19 may make it increasingly difficult for low-income individuals to transition to and obtain postpartum care, and thus may result in a disproportionate increase in adverse outcomes for already vulnerable populations. For example, recommendations by the American College of Obstetricians and Gynecologists (ACOG) to delay the in-person, comprehensive postpartum visit to 12 weeks acknowledge that this decision may negatively affect individuals with publicly funded insurance that expires within 60 days of giving birth [3,4]. Furthermore, these changes may create barriers for postpartum transitions to primary and pediatric care.

Although recent survey studies have helped to elucidate the experiences of pregnant people with prenatal care during the COVID-19 pandemic, little work has focused on the narrative of individuals’ peripartum experiences, especially those of low-income status, as it pertains to their delivery and subsequent engagement with healthcare [5–7]. Identification of barriers to peripartum and postpartum care is necessary to improve engagement and quality of care in a suddenly redesigned maternity care system. Thus, we aimed to elicit the perspectives of low-income postpartum individuals with regards to their healthcare experiences at a single academic center in the United States during the COVID-19 pandemic.

## Methods

This qualitative study was designed to evaluate interactions with the healthcare system experienced by postpartum individuals in the US during the COVID-19 pandemic. This study was conducted among individuals participating within the Navigating New Motherhood 2 (NNM2) study, a randomized controlled trial to determine whether patient navigation can improve postpartum health outcomes for low-income individuals (NCT03922334, registered 4/19/19) [8]. Eligibility criteria for the primary study included pregnant or postpartum individuals with publicly funded prenatal care who received care at the Prentice Ambulatory Care Clinic, which serves low-income populations requiring gynecologic and obstetric care at a single academic medical center in Chicago, Illinois. Participants eligible for the trial were age 16 or older; all participants were either English- or Spanish-speaking. Participants in both the intervention (57.5%) and usual care arms (42.5%) were approached after they completed their postpartum appointment or after they reached 12 weeks from their delivery date, whichever came first, so as to not interfere with the primary aims of NNM2. Approval for this study was received from the Northwestern University Institutional Review Board (IRB). All participants provided written, informed consent prior to their participation; minors were permitted by the IRB to consent for their own study participation without parental consent due to their pregnant or parenting status.

As NNM2 began enrolling participants in the late third trimester in January 2020, early trial participants were newly postpartum at the onset of the COVID-19 pandemic in mid-March, a time when Chicago government and public health officials enacted shelter-at-home orders, subsequently followed by further limitations on non-emergent surgeries and visitor access to hospitals in an effort to limit the spread of the virus. During this first wave, common obstetric guidelines at the participants’ medical institution included a visitor limitation during labor and postpartum (single adult visitor for COVID-19-negative individuals and no visitors for those with COVID-19), neonatal separation for those who were COVID-19 positive, attempts at early postpartum discharge to limit hospital exposure, and exclusive telemedical care for postpartum visits. Furthermore, COVID-19 testing capacity was initially limited, with universal testing for labor and delivery admissions not beginning until April 2020. While guidelines and diagnostic protocols were updated with evolving information on the virus, the majority of participants were subjected to these aforementioned restrictions. Thus, interview questions were developed through team discussions incorporating these recent changes to healthcare delivery with preliminary reports from patient navigators regarding participants’ experiences of the pandemic. Participants were asked open-ended questions that encouraged them to share their experiences with healthcare, whether positive or negative.

From March to June 2020, all NNM2 participants were approached to participate in a single in-depth interview regarding their late pregnancy and early postpartum experiences with healthcare amidst widespread COVID-19 restrictions and changes. Participants were approached after completion of a postpartum visit (or 12 weeks postpartum, as noted above) in order to allow them to engage with their medical providers for postpartum care prior to the interview. Individual interviews were conducted over the telephone by three trained research assistants who were either medical students and/or had master’s level training; all team members had undergone training in the empathic nature of open-ended qualitative interviewing. Interviews used a semi-structured interview guide with open-ended, in-depth queries that allowed participants and interviewers to creatively explore their experiences with prenatal care, labor, telemedicine, postpartum care, and pediatric care. Example topics are noted in Table 1 and the interview guide is included in S1 Appendix. Interviews were completed in the participant’s preferred language (English or Spanish) and lasted from 30 to 60 minutes. Each interview was digitally audio-recorded and professionally transcribed, as well as translated to English if indicated. All participants were compensated for their participation with a $25 gift card.

**Table 1:** Semi-structured interview guide topics.

Topics Addressed
Participants’ ability to prioritize their own health Changes in the participants’ perceptions of their own health Conversations with healthcare providers about COVID-19 Availability of healthcare providers Transition to telemedicine encounters Changes in prenatal and postpartum care Perceived risk of interacting in person with the healthcare system Labor and delivery experience Access to pediatric care

Transcripts were uploaded on Dedoose (www.dedoose.com), a secure web-based qualitative data management and analysis software that facilitates collaborative data exploration and identification of themes. Transcripts were analyzed by a team of four investigators using a constant comparative method [9–11]. Four reviewers individually performed an in-depth analysis of an initial subset of transcripts (n = 5), mapping emerging concepts. A unified codebook was developed after complete team review to allow for organization, negation, and agreement on themes and subthemes. The codebook was subsequently validated and refined with a second subset of transcripts (n = 5), with the reviewers agreeing on eventual saturation of themes. The remaining 30 transcripts were divided and analyzed by two pairs of reviewers, using an ongoing iterative process to ensure consistency with the codebook. Each pair met regularly to ensure agreement on themes and conceptual nodes across reviewers and to resolve any discrepancies. Emerging themes and subthemes are described in the results using illustrative quotations.

In addition, based on the themes, potential solutions to the challenges elucidated by participants were generated using a modified mini-Delphi method, in which members of the research team engaged in an iterative discussion-based process to generate alternatives to current barriers with healthcare utilization.

## Results

Of 50 participants enrolled in NNM2 at the start of the study period, 46 participants were eligible for participation in this qualitative study based on their delivery timing, and all 46 were contacted for interest in participation. Subsequently, 40 participants (87%) were interviewed, with the study team members agreeing upon saturation of themes at this point. Interviews took place on average 10 weeks after giving birth. Fifty percent of participants identified as non-Hispanic Black and 37.5% as Hispanic. Participant demographic characteristics are shown in Table 2.

**Table 2:** Participant demographic characteristics (N = 40).

Characteristic	N (%) or mean (SD)^a^
Age, years	28 ± 5
Weeks since delivery	10 ± 3
Race/Ethnicity	
Non-Hispanic White	1 (2.5%)
Non-Hispanic Black	20 (50%)
Hispanic	15 (37.5)
Language in which interview was completed	
English	38 (95%)
Spanish	2 (5%)
Marital status	
Single/unpartnered	22 (55%)
Married/partnered	17 (42.5%)
Other	1 (2.5%)
Employment^b^	
Full-time work	8 (20%)
Part-time/temporary work	6 (15%)
Unemployed	24 (60%)
Student	2 (5%)
Parity^c^	
0	10 (25%)
1	12 (30%)
2	6 (15%)
3	6 (15%)
4+	6 (15%)

^a^ Data displayed as N (%) or mean ± standard deviation.

^b^ Employment status at the time of participant’s postpartum interview.

^c^ Parity at the time of prenatal recruitment.

Although the interview guide invited responses on either positive or negative experiences, participants’ responses overwhelmingly focused on barriers to care, emphasizing that COVID-related changes in health care delivery were not perceived by this population to facilitate their care. As such, interview responses were organized into three major domains regarding the challenges experienced by low-income postpartum individuals at this time: 1) unanticipated changes in the birth experience, 2) delayed care, and 3) perceived disadvantages of telemedicine. Multiple themes were further developed within each domain. Themes commonly highlighted specific barriers to patient satisfaction with the care received. Exemplary quotations of each theme are found on individual tables.

### Unanticipated changes in the birth experience

The “unanticipated changes in the birth experience” domain addressed both the effects of evolving labor and delivery guidelines on participants’ birth expectations and their perspectives on this unique perinatal period. Themes related to this domain included *uncertainty about COVID-19 status*, *COVID-19 testing*, *separation from newborn*, and *visitor restrictions* (Table 3).

**Table 3:** Perceptions of postpartum participants on alterations to their birth experience.

Unanticipated Changes in the Birth Experience	
Theme	Exemplary Quotations
Uncertainty about COVID status (N = 5)	“When I was pregnant, I was ill myself and again that made me really wonder what was going on at the doctor. Because the doctor had said something about sticking a trachea down my throat if I caught the flu because I didn’t want to get a flu shot and it was really weird because then after this stuff, all of this stuff happened.”
	“I never even knew when was the point where I should get tested. Because I’ve been in the house I didn’t know if there was like more of a risk that I could get infected or not infected, especially with the newborn… Even now I don’t even know…if I should get tested because I possibly had it in the past. I don’t know if my kids should get tested because you know they also got sick, so it’s a lot of like unknowns.”
COVID-19 testing (N = 3)	“They called me a couple of days before my induction date to let me know that they were going to be testing me to see if I was positive for the coronavirus, because even though I had no symptoms, some people could be asymptomatic, so and they were telling me then that if I did test positive they would have moved me somewhere and pretty much that everyone would be in full PPE dealing with me.”
	“Before delivery I was tested and also while I was admitted… I was tested just due to the symptoms of fever that I had but were not actually COVID-related. So at first that was…very frustrating… I just kept getting testing for something just because I have a fever.”
Separation from newborn (N = 4)	“I was actually sick for about like a week or so… but what was suggested is that… I would have a mask on while I was breastfeeding… that was… really weird to kind of have to have like that barrier between me and my newborn. They were also saying that I should try to keep her kind of away from me as much as possible… So that was kind of just kinda weird to have her like really like away from me.”
	“Like seven days or more before I was able to see my baby… That was depressing because it’s been like now difficult to bond with the baby. Breastfeeding her is a fight, and it’s hard to bond with her.”
Visitor restrictions (N = 12)	“When I went in labor I could only have one visitor, so my mom came but my child’s father wasn’t able to come up… That was the most important person that I wanted to be there while I had my baby but they couldn’t… Just felt like more, like you were more alone with it.”
	“I think more so than anything, just people being able to go into the hospital to visit me was a big thing… Like nobody was really welcomed. My 4-year-old wasn’t able to come into the hospital like normal and enjoy the baby like a family, so that was really, really emotionally difficult for her. She cried for the whole three days.”

First, limitations to widespread testing at the beginning of the pandemic left many participants *uncertain about their COVID-19 status* during the perinatal period. For example, one participant experienced severe symptoms consistent with COVID-19 in the weeks prior to giving birth and described her distress over the wellbeing of her fetus and her lack of a diagnosis:

*“Two or three weeks before I gave delivery*, *I ended up really really sick…I came into an ER visit… They didn’t know what it was…I believe it was the coronavirus that I got because it was so so bad*. *I couldn’t taste*, *I couldn’t smell…I was coughing so strongly and being pregnant…I would call my mom in the middle of the night like crying ‘Mom*, *I don’t know what’s wrong with me*. *I don’t know why I’m so sick…It feels like every time I’m coughing it’s putting a strain on the baby…I’m worried*.*’”*

In some cases, individuals with respiratory symptoms during pregnancy retrospectively suspected infection with COVID-19 despite receiving a different diagnosis from their doctor at the time, citing a general mistrust of the healthcare system as the reason behind their persistent reservations (Table 3). Uncertainty about COVID-19 status also manifested as lack of knowledge about indications for testing, both during and after development of COVID-related symptoms. One participant who, along with the rest of her household, became sick shortly after giving birth further emphasized the difficulty of balancing risk of exposure during testing with benefit of knowing her family’s COVID-19 status.

Most participants who did receive *COVID-19 testing* reported it as part of their hospital’s admission process. While some individuals described being informed of testing procedures and potential outcomes ahead of their admission, others neither appeared to understand the indications for COVID-19 testing, nor believed it necessary, indicating the need for clear and effective patient education (Table 3). Individuals who expressed concerns about testing ahead of delivery feared the implications of a positive test result, which, based on the guidelines at the time, would include isolation from their support person and newborn. One participant stated, “We were tested when we came in…So it was kind of nerve-wracking because if I would have tested positive for the virus, I would have had to be there by myself.”

With regards to a potential *separation from newborn*, one of the main worries shared by participants was its influence on breastfeeding (Table 3). One individual who tested positive for COVID-19 emphasized the long-lasting impact of the separation on her relationship with her newborn, attributing her current difficulties with breastfeeding to this disruption. Another participant described the separation from her newborn after testing positive during her induction of labor:

*“I had to be isolated*. *I couldn’t do skin to skin with my baby*. *They only let me see him a split second*, *take a couple pictures from my own phone and then they took him right away upstairs to the isolated nursery*. *I only could see him and connect with him through Facetime…I wanted him to smell me and touch me and stuff and he couldn’t do that*.*”*

Feelings of isolation were central to the birth experience of most participants, including those who did not test positive for COVID-19, and were often attributed to *visitor restrictions*. Many participants described the difficulty of assigning a single companion to be with them during their hospital stay, often having to choose between their partner and their parent (Table 3). Additionally, they reported having less support when making key decisions about anesthetics or birthing positions. For example, one participant described the limitations of having a single visitor, explaining that the person she chose did not have the necessary language skills to meet the needs of her delivery experience:

“*That wasn’t as much supporting as I was hoping it to be because like I also encountered some complications after delivery and*…*my visitor…my mom…doesn’t speak English and the ones that actually are like fluent that understand and speak the language weren’t able to be there to communicate with the doctors…So that was very stressful*.*”*

Multiple participants specifically mentioned the disappointment of not being able to have their doulas present, resorting to video communications with doulas to receive their guidance and support during the birthing process. Children were another common group whose absence was noted by participants (Table 3). In particular, parents with multiple children were limited by restrictions requiring visitors to be at least 18 years old. For example, one participant stated her four-year-old daughter was devastated by not being “able to come into the hospital like normal and enjoy the baby like a family.”

For families whose newborns required prolonged hospitalization in the NICU after birth, visitor restrictions continued to impact their ability to receive emotional support and make collaborative medical decisions. Explaining the effect of restrictions allowing only one parent to visit their newborn at any given time, one participant stated, “that’s like the biggest thing for us right now like parenting-wise just not being able to be there together.”

### Delayed care

In the domain of “delayed care,” participants addressed their perceptions of subtoptimally timed interactions with a variety of healthcare providers. Deferred care was driven by clinical guidelines and by participants’ fear of being exposed to COVID-19 in healthcare settings. Themes related to delayed care included *logistical challenges* and difficulties navigating *postpartum care*, *health maintenance*, and *pediatric care* (Table 4).

**Table 4:** Perceptions of participants on healthcare delays during the COVID-19 pandemic.

Delayed Care	
Theme	Exemplary Quotations
Logistical challenges (N = 14)	“[The hospital] sent an email saying ‘Hey guys, we are no longer doing appointments. We can do them over the phone’… So I went ahead and sent an email back. I never received anything back from them. So I guess maybe there was a whole bunch of people. I don’t know. But I’m still waiting to see to talk to somebody.”
	“Being that most clinics are… only taking clients for emergency purposes, sometimes it’s hard to reach someone by phone or to get a return phone call back, so that process of having to wait with unanswered questions can be a little bit uneasy.”
Postpartum care (N = 14)	“I wanted to get my Paragard checked out because I had been pregnant with that before… so I just was wanting to make sure it’s in the right place… because there’s a way I can get pregnant if it ain’t in correctly but they put it in right after I delivered… Now I gotta wait until I can get in there and see the OB/GYN.”
	“It [COVID] did affect a lot because…I was gonna get my birth control and of course I couldn’t do that…I’m on birth control right now but not the one I wanted.”
Health maintenance (N = 8)	“They don’t really see you unless you have an emergency, so it’s like sometimes it’s just like you put your health on like the backburner.”
	“I am concerned about managing it [diabetes] during this time. I’m unsure if I will be able to see my endocrinologist because a lot of doctors are rescheduling because of COVID and I’m concerned about my blood sugars and being able to keep it under control during this pandemic is very hard without being able to exercise.”
Pediatric care (N = 20)	“She had two-month shots due ‥ [she’s] behind on her shots. I don’t know how much she weighs. I don’t know like where on the chart she is in terms of her growth and development.”
	“She hadn’t had her shots. Like she just got her shots a week and a half ago and…she should have gotten her shot in March…They’re not seeing any babies right now. They just want us to stay in the house. Like I just want her to have the same options.”

The sudden cancellation of healthcare appointments at the onset of the pandemic created multiple *logistical challenges* that impacted participants’ ability to receive timely care. After being informed of their appointment’s cancellation, many individuals described a lack direction from their doctor’s office when trying to reschedule the visit or transition to telemedicine (Table 4). In particular, this posed a greater difficulty for individuals accustomed to making future appointments when in person at their clinic. Additionally, many participants cited unusually high call volumes, long wait times, and limited office staff as barriers to prompt communication with healthcare professionals, with one participant stating:

*“It was very difficult*, *and it was a week of me calling and…not being able to get through…my primary being so busy…with COVID and it may have been also her working with a smaller staff*. *I was on the phone for days just to get through to be able to make an appointment*.*”*

Although the majority of participants were able to obtain some form of *postpartum care*, many participants worried about the delay of their physical examination after giving birth, commonly citing concerns about prolonged bleeding, intrauterine device positioning, and incision healing (Table 4). Similarly, others emphasized having to wait for future appointments to receive their contraceptive of choice. Referring to the postpartum visit, one individual said, “I didn’t have it at all… No one ever called me back… So I didn’t, I never got that care… I’m still bleeding.” As an example of the importance of timely care in the postpartum period, another individual noted having to rely on family members and online resources for breastfeeding guidance after the indefinite cancellation of her appointment with a lactation specialist.

For many participants, *health maintenance* also brought its own challenges, with one participant detailing the difficulty of care coordination during the pandemic:

*“I was transitioning from my OB/GYN doctors* … *to a primary care doctor and unfortunately because of the timing I was not able to see a primary care doctor so there’s really no one to write a prescription for my insulin right now and that is concerning for me*.*”*

Other individuals also described the difficulty of establishing care with a new provider during the pandemic for non-emergent health problems, stressing the negative impact these conditions can still have on their well-being (Table 4). Participants described interruptions to essentially all forms of preventive care, from cervical cancer screening to blood tests. In some cases, participants themselves chose to delay these measures to avoid unnecessary exposures to COVID-19. For individuals with chronic health conditions, challenges to medication access emerged as the main consequence of delayed care, often resulting from a lack of continuous care outside of prenatal appointments or from a lack of guidance on prescription refill in this new context. After the death of her newborn, one participant stated, “I wanted to get back on my medication [for bipolar disorder]. I wasn’t able to do that, I’m not able to see the doctor I need to see.”

When reflecting on their experiences with *pediatric care*, individuals often expressed concern over the delay of their newborn’s vaccination schedule (Table 4). In some cases, this delay was dictated by their pediatrician’s safety measures. In other cases, participants chose to delay the vaccines because they believed the potential exposure to COVID-19 in the pediatrician’s office posed a higher, immediate risk to their newborn. Several participants reported that their newborn’s pediatric appointments continued to be held in person, with longer gaps between appointments. However, individuals with older children often saw their appointments re-scheduled or canceled, which required parents to advocate for their care in order to obtain the desired medical encounters. Dealing with her son’s earache, one participant explained, “I begged the nurse…They were able to squeeze him in, but at that time it was just like too much time…so I kinda like improvised on my own.” Unable to rely on previous experience, first-time parents shared the negative impact of limited contact with their child’s pediatrician, such as the individual who said:

*“It’s actually been kind of hard because it’s like I haven’t been able to take her to any other doctor’s appointment since the first one ever…I don’t know what is required for a newborn…I’m a new mom*, *I’ve never done this before…I’m using people to kinda help me through everything because like questions I have for the doctors*, *it’s so hard to kind of get a hold of them too*.*”*

### Perceived disadvantages of telemedicine

Perceived disadvantages of telemedicine encompassed both lived and potential difficulties for participants receiving care in a solely virtual setting. Themes included *technical difficulties*, *difficulties establishing rapport with providers*, *incomplete health information*, and *lack of resolution to healthcare problems* (Table 5).

**Table 5:** Perceptions of participants on telemedical encounters.

Perceived Disadvantages of Telemedicine	
Theme	Exemplary Quotations
Technical difficulties (N = 5)	“It’s been difficult to have those appointments in person. Instead they’re like over the phone or video and a lot of the times it’s like I have trouble with video connection. So it’s very stressful.”
	“Doctors are only doing like Facetime calls. I’m not the greatest with like computers and all that stuff. I just know how to do like the basic stuff. So the doctor wanted to do like a Facetime call or something. I just didn’t know how to get it connected and all that.”
Difficulties establishing rapport with providers (N = 8)	“I like to see my doctor face to face. I feel like body language… is a total way to totally understand what’s actually going on instead of just hearing my voice over the phone. So that was one thing that was uncomfortable for me.”
	“I feel like maybe it’s better in person because when you can see somebody’s facial expression or how they react to a certain question or comment, that probably tells you more about the question you’re asking than someone’s actual answer. I think maybe a little bit changed because of that.”
Incomplete health information (N = 10)	“I received this call from one of the nurses or doctors, I mean it was good but at the same point it was like okay, like what’s the point of this? Because they were like … ‘Touch your baby’s head and like just can you feel like that like an empty space like right in between on the baby’s head. Like do you feel it?’ I’m like yeah. ‘Oh okay, well, is it super deep?’… Those are the type of things that it’s like I would rather have the appointment in person.”
	“The doctor… was actually pretty thorough. It’s just… it’s only so much that I’m able to understand about what the doctor is giving me information about because I’m not a medical professional… so sometimes seeing different things, it helps… from you know when you’re in the office, the doctor might have access to more reference or literature, pictures. So it’s easier to understand.”
Lack of resolution to healthcare problems (N = 6)	“[My baby] had a hernia or what I believe was a hernia and the doctor told me like ‘Yeah, I think it’s one too but I want to wait a few weeks until I can really look at it.’ We did the FaceTime video… but you couldn’t really look at it so now I still have to wait…It’s kinda like pointless.”
	“It’s very different because it feels as if I’m just having a conversation but I’m actually like not being checked physically. So it’s different to see like a result like an outcome result to the issue that I’m having… It’s just as if I’m doing like the same home remedies, like just continue taking my prescription basically.”

The abrupt transition to telemedicine required participants to quickly adapt to a new form of healthcare delivery, often thwarted by *technical difficulties*. Many participants reported being primarily affected by an unreliable internet connection, which limited their ability to participate in encounters over video:

*“My cell phone*, *it has limited data*, *so I’m not really able to video chat much*. *When I do it with my family*, *I do it a few times and that’s pretty much it*. *Then it’ll start like freezing or coming on saying low data*. *So just not being able to have like the actual access to kind [of] do it and video chat… it’s hard*.*”*

Despite having internet access, others mentioned that limited technological skills and support were barriers to scheduling telehealth appointments and receiving telemedical care (Table 5). Citing the quick turnover of telemedical appointments, a participant reported being unable to speak to her provider after failing to answer her phone, despite returning the call. One Spanish-speaking participant shared her experience using an interpreter during the telephone encounter, stating the difficulties of being heard and of effectively communicating with her provider on a conference call.

In some cases, participants stressed the strangeness of having a doctor’s appointment in the privacy of their own homes, likening the interaction to a conversation as opposed to a medical visit. Many individuals reported *difficulties establishing rapport with their providers* over telemedicine:

*“I had to do a phone call visit instead of an office visit*, *so that affected me*. *I feel like sometimes you can bond with your doctors a little more if you’re in their space and you talk to them in person and stuf…*.*You can build a relationship with them kind of better*.”

Without an opportunity for small talk prior to addressing the reason for their visit, participants often described the encounters as “awkward” or “uncomfortable,” particularly when unable to see their providers over video in appointments that were solely over the telephone (Table 5). Multiple participants emphasized the importance of body language as a communication tool, with one individual explaining that doctors might be able to “see” a concern that a postpartum mom is unable to express herself (Table 5). Acknowledging that some individuals might not like to be on video, one participant proposed offering patients the option to speak over the phone or to have a video call as a way of giving appointments “a person-to-person touch.”

Participants commonly attributed their dissatisfaction with telemedicine to *incomplete health information*. For some participants, this meant that providers were unable to use objective data to assess their health, having to rely on descriptions of concerns instead of physical examinations (Table 5). This was especially challenging for those who lacked the medical equipment necessary to monitor medical conditions, such as the participant who stated:

*“I would prefer… a person-to-person visit*… *I do suffer from like high blood pressure during the pregnancy and they asked me if you know like I’m checking it and I’m like you know I don’t know where I would be able to check it or what to do… It was a little tough to do it over the phone*.*”*

Participants emphasized the difficulty of relaying clinical information, especially concerning their newborns, due to a lack of knowledge of medical terminology. Even when communicating about their own health, some struggled to find the adequate words to phrase their concerns without relying on gestures. Communication challenges not only centered around conveying information but also around receiving it, with one participant reporting the increased challenge of understanding the provider over the phone without the assistance of printed handouts or diagrams (Table 5).

Partly because of these limitations, some participants reported *a lack of resolution to their healthcare problems* after telemedicine encounters (Table 5). One participant expressed the frustration of having to wait for her health concern to worsen in order to warrant in-person care:

*“I don’t really feel like doctors’ appointments should be over the phone*… *Like I’m trying to show them something’s wrong and it’s like ‘Oh*! *just keep on being on the lookout*, *like if it gets worse then call us*.*’ Instead of them like taking action like right away*.*”*

Furthermore, several individuals reported ending their virtual postpartum appointments without full reassurance of their wellbeing, having been unable to complete a physical exam. As a result, they reported planning to have a more thorough encounter once in-person appointments became available. In one case, a participant shared canceling her postpartum appointment after it was changed into a telemedical encounter, saying this was “just a waste of my time. Like I need to physically be seen in person.”

## Discussion

Our study offers novel perspectives on barriers to healthcare interactions in the US during the COVID-19 pandemic among low-income, predominately minority individuals, a population that has disproportionally borne higher rates of COVID-19 mortality and infection in Chicago and much of the country [12–15]. We identified that *unanticipated changes to the birth experience*, *delayed care*, and *perceived disadvantages of telemedicine* heavily influenced participants’ ability to remain engaged in their own healthcare. Important lessons can be taken from these interactions in the first wave of the pandemic to ensure the delivery of equitable healthcare for low-income populations in the current context and beyond.

Few studies have explored the experiences of low-income individuals as they navigate healthcare in the postpartum period during the COVID-19 pandemic. In a recent study of patients’ experiences with obstetric care, participants reported being forced to alter their original plans of having family members and/or doulas present during their delivery [6]; our findings on the effects of *visitor restrictions* similarly highlighted the impact of diminished social support in labor. Postpartum survey respondents also listed access to well-child and postpartum visits as major concerns, paralleling the narratives established within our *pediatric care* and *postpartum care* themes. Further, a study by Karavadra et al. surveyed individuals who were pregnant or had recently delivered in the United Kingdom (UK), identifying impersonal care and internet connectivity problems as barriers to effective antenatal virtual consultations during the pandemic; their findings aligned with our themes of *technical difficulties* and *difficulty establishing rapport with providers* as *perceived disadvantages of telemedicine*. Participants in the same study were also torn between avoiding healthcare settings in the antenatal period to limit COVID-19 exposure and worrying over their health as a result of limited in-person appointments and ultrasounds, with this finding resembling our domain of *delayed care* [7]. Another study of users of a pregnancy and parenting app in the UK also found pregnant and postpartum individuals felt reduced support from health care professionals, with many having appointments delayed or cancelled. Furthermore, participants expressed concern over receiving suboptimal care via telemedicine, leading to anxiety over the health of their children [16]. Despite large differences in demographic characteristics, health systems, and timing of inquiry throughout the peripartum period, the findings in these studies strongly align with many of the experiences shared by participants in our study, emphasizing the widespread need for care optimization during the pandemic.

With the rapid expansion of telehealth services, optimization of telemedicine care remains an important need. The removal of reimbursement restrictions by the US DHHS and the Centers for Medicare & Medicaid Services has promoted the widespread adoption of this technology [17]. Prior work has explored and emphasized the benefits of implementing telemedicine in prenatal care [18,19]. Outside of the current pandemic, this technology offers a novel way to maintain engagement in care for individuals who are homebound or who live in rural or inaccessible urban areas. Furthermore, telemedicine could serve as a facilitator to improve rates of completion of postpartum visits by providing increased flexibility to individuals who might still be recovering from their birth experience and/or hesitant about leaving the household with a newborn and by allowing providers to more frequently monitor patients at higher risk of adverse outcomes. However, despite theoretically being meant to obtain the most assistance from this intervention, low-income participants in our study rarely highlighted benefits of this technology. We were unable to identify themes centered on benefits, with only brief mentions of reductions in commutes and mitigation of social anxiety emerging as potential upsides to an otherwise challenging care model. The wide benefits that can be derived from telemedicine were largely overshadowed by the obstacles reported by nearly all participants in this low-income population. This discrepancy might be attributed to the sudden adoption of this technology, which allowed little time for adequate training of providers and likely impacted patients’ perception of its efficacy, and to the technology’s intrinsic limitations to physical examinations. A recent study by Ukoha et al. further explores barriers to equitable implementation of telemedicine and establishes methods for addressing said barriers at multiple levels, emphasizing the importance of ensuring that low-income and underserved individuals also benefit from this technology [20].

### Potential solutions

Understanding the challenges experienced by low-income pregnant and postpartum people as the COVID-19 pandemic evolves is critical to informing new guidelines that limit further widening of existing health inequities. Focusing on individual-level perspectives on the efficacy and quality of care, we developed patient-centered solutions that may mitigate some of the limitations to care reported by this population during the pandemic (Table 6). Based on our findings in the domain of *delayed care*, we propose an emphasis on shared decision making for healthcare services, particularly for cases that providers or patients feel might not be effectively addressed over telemedicine. Essential to this process are transparent conversations on the medical rationale for in-person versus telemedicine appointments. For patients with chronic health conditions, we propose providing patients with clear and comprehensive instructions and/or necessary equipment, such as blood pressure cuffs or scales, to effectively monitor their health at home. Having consistent access to objective data that can be addressed with their medical providers via telemedicine may help patients feel in control of their health status during a time of uncertainty (Table 6).

**Table 6:** Potential solutions generated from patient-reported challenges to healthcare access during the COVID-19 pandemic.

Theme	Subtheme	Potential Recommendations^d^
**Delayed Care**	Logistical challenges	For patients with chronic health conditions such as hypertension and diabetes, offer clear and comprehensive patient instructions and resources for obtaining measurements of necessary values at home. Practice shared decision making, involving patients in benefit vs. risk considerations regarding healthcare services that cannot be effectively provided over telemedicine. Prioritize patients with complex health problems for in-person appointments. Provide patients with a list of free exercise resources that can be effectively completed at home with limited equipment. Educate parents on vaccination schedules and developmental milestones to reassure them that the child is receiving the necessary care.
	Postpartum care	
	Health maintenance	
	Pediatric care	
**Perceived Disadvantages of Telemedicine**	Technical difficulties	Offer patients a list of resources (e.g., videos, infographics) and/or virtual trainings on using the appropriate telemedicine platform ahead of their scheduled appointments. Encourage and provide instructions on the use of electronic medical record-connected patient portals. Inform patients of the possibility of having appointments via phone call if the patient doesn’t have the technological means to support a video call (i.e no cell phone data, no camera). Provide patients with instructions and contact information to use in the event of a disconnected call or other technical difficulties during a telemedicine appointment. Offer flexibility for cancellations or rescheduling, particularly for populations with greater technology access barriers.
	Difficulties establishing rapport with provider	Develop effective communication strategies to prioritize rapport building during telemedicine appointments, particularly for patients who are establishing care with a new provider. Offer patients the opportunity to have video appointments so that they are able to see their providers and engage in non-verbal forms of communication.
	Incomplete health information	See “Health maintenance” regarding clear instruction on home monitoring for health status. Offer patients educational aids (e.g., handouts, diagrams) during or after telehealth encounters to address health literacy barriers and improve patient understanding. Engage in shared decision-making regarding options for in-person care when it is strongly desired by the patient or provider. Practice empathy and listening skills to effectively determine patient needs and provide reassurance. Explain the medical rationale for a telemedicine vs. in-person patient appointment.
	Lack of resolution to healthcare problems	

^d^ Note that potential recommendations to address the sub-themes of uncertainty about COVID-19 status, COVID-19 testing, visitor restriction, and newborn separation are not described, as clinical care for these issues is based on recommendations from professional and governmental organizations.

Furthermore, we propose the development of effective communication strategies to prioritize rapport-building during telemedicine appointments, particularly for patients who are establishing care with a new provider (Table 6). A good patient-provider relationship is essential for telemedicine, particularly when providers are only able to rely on information reported by patients and not on additional physical examination or labs. Additionally, we recommend providing patients with resources and/or virtual trainings on the use of institutional telemedicine platforms ahead of their appointments and with clear instructions and contact information for use in the event of technical difficulties, acknowledging that many individuals might not have reliable access to internet services. Lastly, we recommend modifying traditional patient education tools (handouts, diagrams) to formats that are compatible with telemedicine so that they are readily available to patients throughout or after encounters in order to improve patient understanding and autonomy.

### Strengths and limitations

Strengths of this investigation include centering on an underrepresented and underserved population at various stages of their peripartum period coinciding with the first wave of the COVID-19 pandemic in the US. Participants were primarily low-income non-Hispanic Black and Hispanic individuals, capturing minority groups that historically and presently experience increased barriers to healthcare access. Additionally, this study is one of the first to provide the perspectives of low-income postpartum people in the US, as the majority of the current investigations on the topic have focused on prenatal care among individuals of a higher socioeconomic status in countries with different healthcare systems. This study also allowed participants and interviewers to create conversational narratives about the outcomes or quality of participants’ healthcare interactions during the pandemic. The wide range of healthcare topics and the depth of conversation thus allowed us to frame the challenges that low-income individuals face as they navigate the many facets of healthcare taking place in this important life stage.

However, this study has limitations. First, participants gave birth at a single urban academic medical center in the initial months of the pandemic, a time when public health guidelines were quickly evolving. We now have a better understanding of the virus and healthcare delivery during the ongoing pandemic. As such, guidelines have changed. Although the central themes remain relevant, our findings require interpretation to the current context and, as is expected for qualitative research, may not be generalizable. Secondly, while the study population captures a significant percentage of non-Hispanic Black and Hispanic participants, there’s a limited number of White participants. Thirdly, interviews were carried out over the telephone, potentially missing body language and visual cues that could have helped guide the conversation. Additionally, the potential effect of the patient navigation intervention itself is unknown, although the persistence of challenges for individuals in both arms of the trial suggests navigation did not fully ameliorate the barriers. Lastly, participants were not explicitly asked to offer potential solutions to their barriers with healthcare interactions. An important area for future exploration is to generate solutions that best ensure patient participation and satisfaction and that emphasize the priorities of low-income populations.

## Conclusion

In summary, identification of barriers to healthcare interactions for low-income peripartum individuals is critical to mitigate new or heightened limitations of care that surfaced with the COVID-19 pandemic. The sudden move towards prenatal and postpartum telemedicine, the visitor restrictions imposed during hospitalization, the potential effects of being ill with COVID-19, and the overall concerns over transmission of the virus exacerbate the physical and mental health challenges and fragmentation of care that already serve as common reasons for suboptimal healthcare during the postpartum period and that disproportionally affect low-income populations [1].

Although public health guidelines and clinical care have evolved since the beginning of the pandemic, particularly with the development of vaccines against COVID-19, new variants of the virus have emerged which continue to alter the way patients engage with healthcare, ensuring that many of our findings remain relevant in the current context of the pandemic. However, challenges and disruptions in care experienced during the pandemic by low-income, underserved populations have the potential to affect the health and well-being of many individuals, even in a post-COVID society. For instance, interruptions in vaccination schedules and preventive screenings delineated by participants within the domain of the “delayed care” could have long-term public health implications if the burden of rescheduling said encounters is placed on patients alone. Additional support, such as in the form of patient navigators, will likely be required to ensure that care delayed during the pandemic is eventually received by patients in more vulnerable groups. Furthermore, as we begin to explore the long-term psychological effects and potential trauma associated with living and parenting through a pandemic, there is value in better understanding the experiences of postpartum individuals during this critical time.

Optimizing hybrid models of telemedicine and in-person care to ensure equitable care should remain a priority as COVID-19 continues to alter traditional models of healthcare delivery. The lessons learned in the first wave of the pandemic are integral to devising well-established protocols of care that can be quickly adapted to present and future circumstances, whether these are epidemics or natural disasters. Furthermore, the use of telemedicine can help bridge long-standing obstacles to healthcare access in underserved populations to ensure that they receive adequate and reliable medical care. If we are able to capitalize on these benefits while reducing perceived disadvantages and the threat of inequitable implementation, telemedicine might serve as a critical tool to improve the health of this population in the postpartum period, a critical time often perceived as a “fourth trimester” [1].

## Supporting information

S1 Appendix (DOCX)
