# Supplementary material for: Postpartum during a pandemic: Challenges of low-income individuals with healthcare interactions during COVID-19
Source: PLoS One. 2022 May 24;17(5):e0268698. doi: 10.1371/journal.pone.0268698 (PMC9129029; doi:10.1371/journal.pone.0268698)
Supplement: S1 Appendix — (DOCX) [file pone.0268698.s001.docx]

**S1 Appendix. Interview Guide**

**Navigating New Motherhood 2: COVID-19 Interview Questions**

Hello! Thank you for participating in today’s interview. You have been asked to participate because you are enrolled in Navigating New Motherhood. Our Navigating New Motherhood participants are experiencing pregnancy and early motherhood during an unusual time in history, due to the coronavirus pandemic. We understand that this pandemic, which is referred to as COVID-19, may affect your experience of pregnancy and motherhood. We also understand that this may be a particularly important time for having increased support from the health care system. For all these reasons, we are interested in understanding more about pregnancy and parenting during this pandemic. I will ask you some questions on these issues during our call. If any questions make you feel uncomfortable, you may choose to skip them. If, at the end of this call, you would like further resources, we would be happy to connect you with your physician team.

Before we begin, I just want to remind you that there are no wrong answers. We are only trying to get a better understanding of your experience during this time. In addition, what we discuss today will not be shared with your healthcare provider or any clinic staff. Our conversation will be digitally recorded so that I do not miss anything.

**POSTPARTUM PARTICIPANTS:**

**First, we will talk about your experience of this epidemic in general, but particularly from the perspective of already being or becoming a mother. Tell us, in your own words, how COVID-19 has affected your experience of pregnancy, parenting, or your anticipation of parenting a new baby. We are interested in all of your concerns regarding these issues.**

- How has the current crisis affected your social interactions?
  - Have you actively tried to reduce the amount of visitors that you have over to your home?
  - How has the current crisis affected your ability to see or spend time with family, friends, and neighbors?
  - How are you receiving social support?
  - Do you have other children at home?
  - How has the current crisis affected your ability to receive help from your family, friends, or neighbors? For example, help with childcare, household chores, etc.?
- How has the current crisis affected your ability to go out of your home?
  - How often are you leaving your home, and for what activities?
- How has the current crisis affected you financially?
  - Has it had an impact on your (or your family’s) job, income, expenses, rent, or savings?
  - How do you imagine the pandemic will affect your ability to provide resources to your growing family in the coming months?
- How has the current crisis affected your mood and emotional well-being?
  - Can you tell us more about how you are coping with being homebound, especially as a new mother?
- How has the current crisis affected how you perceive your health?
  - Have you noticed any changes in your health?
    - For example, in your quality of sleep, energy, stress, or any postpartum issues.
  - How has the current crisis affected your ability to prioritize your health?
    - For example, taking your medications, measuring your blood sugar, exercising, etc.
- Have you and your doctor talked about COVID-19?
  - What information would you like to receive from your doctor?
  - Do you know who to contact if a family member is feeling sick?
  - Have you talked to any healthcare providers, other than your doctor? What kind of topics have you talked to them about?
  - How has the current crisis affected your ability to communicate with healthcare providers?
  - Have you transitioned to receiving care by phone or video, also known as telemedicine?
    - If yes, tell us more about your experience interacting with providers through calls or videos.

**Next, we will talk about the experience of COVID-19 as a new mother.**

- How much do you think you are at risk of COVID-19?
  - Tell us why you feel you are at a higher (or lower) risk?
  - How much do you worry about passing the virus to your baby?
- Did the current crisis affect your prenatal care? How so?
  - If you received less in-person prenatal care than usual, how did you feel about this?
- Did the current crisis affect your delivery? How so?
  - Tell us about any unexpected changes with labor, timing of delivery, or your plans for pain control.
  - Tell us about any other unexpected changes in your labor and delivery process.
- Next, tell us your level of concern regarding your recent delivery at a hospital.
  - Tell us your level of concern regarding potential exposure to the virus in the hospital.
  - Tell us your level of concern regarding being separated from your baby after delivery.
  - Tell us about your hospital experience with regards to having visitors and support with you.
- How has the current crisis affected your postpartum plans?
  - Tell us about your concerns with regards to receiving the care you need during this time.
  - What aspect of postpartum care are you most concerned about not receiving (or being delayed) due to the current crisis?
  - Have you had a postpartum visit already?
    - If so, was it by phone or in person? How did you feel about whether it being (by phone / in person)?
- How has COVID-19 changed your plans to breastfeed? How so / why not?
  - Tell us your level of concern regarding COVID transmission to the baby during breastfeeding.
  - Do you have any other special concerns regarding COVID and breastfeeding?

**Since you are already parenting a newborn, we are also interested in your parenting experience during this crisis.**

- How has the current crisis affected your ability to buy baby supplies (diapers/formula/etc.)?
- How has the current crisis affected the experience of receiving care for your new baby at the pediatrician?
- IF MULTIPLE CHILDREN:
  - How has the current crisis affected childcare for your other children?
  - How has having your other children home affected taking care of your newborn?

**FOR NAVIGATED PARTICIPANTS: Finally, we know that you have been working with {} as your navigator during the study. We would like to know if having a patient navigator has affected your health and well-being during this crisis.**

- How has having a navigator impacted you during the current crisis?
  - Have the navigators helped connect you to your doctors, find resources in your neighborhoods, provide emotional support, or do any other activities?
- How often have you communicated with your navigator during the current crisis: same, more than usual, or less than usual?
  - (If more or less) Why do you think this is?
- What can your navigator do to help you during these times?

**Do you have any final comments or thoughts about these topics?**

Thank you for your time! Your experience may help others get through this difficult period
